# Immune Response to Childhood Vaccination in Vertically Infected People Living with HIV: A Long-Term Evaluation

**DOI:** 10.3390/vaccines13080871

**Published:** 2025-08-16

**Authors:** Annachiara Zin, Elisa Barbieri, Giulia Brigadoi, Andrea Berlese, Lorenzo Chiusaroli, Daniele Mengato, Andrea Francavilla, Carlo Giaquinto, Daniele Donà, Osvalda Rampon

**Affiliations:** 1 Division of Pediatric Infectious Diseases, Department of Women’s and Children’s Health, University of Padua, 35128 Padua, Italy; 2 Hospital Pharmacy Department, University Hospital of Padua, 35128 Padua, Italy; 3 Unit of Biostatistics, Epidemiology and Public Health, Department of Cardiac Thoracic Vascular Sciences and Public Health, University of Padua, 35131 Padua, Italy

**Keywords:** HIV, children, immunization, vaccination

## Abstract

Background: Despite virological suppression through antiretroviral therapy (ART), people living with HIV (PLHIV) may exhibit inadequate immune responses to vaccination, placing them at continued risk for preventable infectious diseases. Evidence regarding the durability of vaccine-induced immunity in PLHIV with vertically acquired infection remains limited. Methods: We conducted a cross-sectional observational study to evaluate humoral immunity to routine childhood vaccines in a cohort of PLHIV with perinatally acquired infection. Antibody titers against diphtheria, tetanus, measles, mumps, rubella, varicella, and hepatitis B (HBV) were retrospectively assessed via serological testing and review of medical records. Seroprotection rates were analyzed at predefined intervals following the completion of the primary immunization schedule. Multivariate analysis was used to explore potential predictors of long-term immune response. Results: A total of 85 individuals were included. Two years after completing the primary vaccination series, seroprotection rates were as follows: diphtheria 71%, tetanus 79%, measles 79%, mumps 67%, rubella 87%, and varicella 54%. Five years post-vaccination, 50–70% of participants maintained protective antibody levels, declining further to 50–58% after ten years. By twenty years, protective immunity dropped below 30% for all antigens except rubella (47%). HBV vaccine responses were notably poor, with only 60%, 37%, 24%, and 7.5% retaining protective anti-HBs titers at 2, 5, 10, and 20 years post-immunization, respectively. Time elapsed since vaccination was the sole significant predictor of seroprotection across all vaccines. Conclusions: In this cohort of vertically infected PLHIV, vaccine-induced immunity was suboptimal and declined markedly over time compared to the general population. These findings highlight the need for tailored immunization strategies, including timely boosters and regular serological monitoring, to maintain long-term protection in this high-risk group.

## 1. Introduction

HIV infection continues to represent a major global health burden, affecting approximately 39 million individuals worldwide, including an estimated 1.5 million children—most of whom acquire the virus via vertical transmission from mother to child [1]. Since the advent of antiretroviral therapy (ART) in 1996, the natural history of HIV has been dramatically altered, enabling people living with HIV (PLHIV) to achieve life expectancies approaching those of the general population, thereby reframing HIV as a chronic, manageable condition [2,3].

Vaccination plays a critical role in the long-term clinical management of PLHIV and is strongly recommended across all age groups. Despite partial immune reconstitution achieved through ART, PLHIV, particularly children, remain susceptible to several vaccine-preventable diseases [4]. These include not only common viral pathogens such as measles, varicella, and influenza, but also invasive bacterial infections caused by *Streptococcus pneumoniae*, *Haemophilus influenzae* type b, and *Neisseria meningitidis* [5]. Additionally, HIV-HBV co-infection accelerates liver disease progression, increasing the risk of cirrhosis, liver failure, and hepatocellular carcinoma [6]. Nevertheless, vaccination coverage among HIV-positive children remains suboptimal, even in Europe [4].

In light of the specific clinical and immunological considerations of this population, tailored immunization schedules are recommended to optimize both safety and efficacy. Most vaccines are considered safe for PLHIV; contraindications are generally limited to live attenuated vaccines in the context of severe immunosuppression. Measles, Mumps, Rubella, and Varicella (MMRV) vaccines are considered safe in the absence of severe immunosuppression (i.e., CD4 count ≥ 200 cells/mm^3^ or ≥15% if over 5 years old) and during ART. Conversely, other live attenuated formulations, such as intranasal influenza vaccines, remain contraindicated regardless of immune status [4,5,7,8,9,10].

Vaccine-induced immunological protection is frequently suboptimal in PLHIV [11,12]. Numerous studies have demonstrated that HIV-positive children develop lower and less durable antibody responses to vaccinations compared with their HIV-negative counterparts. This disparity is most evident when immunization occurs during periods of immunosuppression or prior to the initiation of ART, but it may persist even after immune reconstitution has been achieved through ART [11,13,14,15]. While ART significantly improves vaccine responsiveness [11,13], evidence indicates that immunity may not be long-lasting, as antibody levels decline more rapidly in PLHIV [11,12,16] than in the general population, even among those on ART.

Several studies have attempted to identify the immunological and clinical determinants of vaccine responsiveness following ART initiation. Parameters such as CD4+ T-cell count at the time of vaccination, CD4+ nadir, viral load control, and age at revaccination have been investigated; however, findings remain inconsistent due to methodological and population heterogeneity across studies [11]. Early initiation of ART, particularly within the first year of life, has emerged as a consistent predictor of improved vaccine-induced immune responses [4,13,17], and is strongly associated with long-term maintenance of functional humoral memory [18].

In this context, our study aimed to characterize vaccine-induced immunity in vertically infected PLHIV by evaluating seroprotection rates following primary immunization, their durability over time, and the clinical and immunovirological factors associated with a more robust and sustained immune response.

## 2. Materials and Methods

### 2.1. Study Design and Population

A retrospective, cross-sectional observational study was conducted including data between 1 January 2004, and 31 October 2023, to evaluate the serological response to routine childhood vaccinations in a cohort of vertically infected PLHIV followed at the Pediatric Infectious Diseases Clinic, Department of Women’s and Children’s Health, University Hospital of Padua.

All PLHIV who had received at least one vaccine dose—documented in medical records and, when available, vaccination certificates—and who had serological data collected during follow-up visits as part of standard care were included.

The study protocol was approved by the local Ethics Committee (Prot. CET 407n/AO/23 of 14 September 2023). Written informed consent was obtained from all participants and their legally authorized representatives.

This study follows the Strengthening the Reporting of Observational Studies in Epidemiology (STROBE) guidelines [19].

### 2.2. Data Collection and Variable Definition

When available, vaccination certificates updated to 2023 were reviewed to collect information on the primary vaccination series and any booster doses, categorized by vaccine type. A vaccination series was considered complete according to the CDC-recommended immunization schedule [20].

Seroprotection data were collected by reviewing available serology results from routine examinations conducted during follow-up visits.

Serologies for which there was no corresponding vaccine dose were excluded. PLHIV with concomitant chronic hepatitis B infection were excluded from HBV serological analysis.

Specific IgG titers for each vaccine were recorded. First, we documented the most recent serology available up to October 2023. Second, for each PLHIV, we selected any available serological value at five-time points following the last vaccine dose of the primary series (i.e., 2 years, 5 years ± 1 year, 10 years ± 2 years, 15 years ± 2 years, and 20 years ± 2 years).

Demographic, immunovirological, therapeutic (including the ART initiation date), and vaccination-related data were retrospectively extracted from medical charts during routine follow-up visits. Specifically,

- CD4+ T-cell counts were retrieved for both the year 2023 and at the time of the last of the primary series.
- HIV viral load was recorded for both the year 2023 and at the time of the last dose of the primary series.
- HIV disease stage prior to antiretroviral therapy (ART) initiation and in 2023 was determined according to CDC criteria.

According to standard clinical practice, CD4+ counts were measured by flow cytometry and expressed as cells/mm^3^. HIV viral load was quantified using RT-PCR and expressed in copies/mL; values below 50 copies/mL were considered undetectable. For analysis, viral load values were categorized as <50 copies/mL, 50–200 copies/mL, and >200 copies/mL.

### 2.3. Serological Assays

The serological results were categorized as protective or non-protective (incomplete/doubtful protection or negative) based on the cut-off values indicated in the laboratory records of the Padua University Hospital Laboratory. Analyses were performed using commercial methods: enzyme linked immunosorbent assay (ELISA) for diphtheria, tetanus, and measles, chemiluminescence for mumps, rubella, varicella, and hepatitis B. Reference ranges for interpreting quantitative results are provided in the Supplementary Material [4].

A literature search was conducted to identify seroprotection prevalence data for the general (non-PLHIV) population at the same time points used for comparison with our cohort [21,22,23,24,25,26,27,28,29,30].

### 2.4. Statistical Analysis

Continuous variables, after assessing the normality of the distribution, were reported as either mean (±standard deviation) in case of a normal distribution, or as median and interquartile range (IQR) for non-normally distributed variables. Categorical variables were reported as absolute frequencies and percentages. Seroprotection by vaccines of interest was evaluated by dividing the number of subjects with protective serological results by the total number of subjects with serological results at the different time points considered.

Various statistical tests were used to compare independent variables (clinical–immunological factors) with the primary vaccination outcome at different time points. For normally distributed continuous variables, Student’s *t*-test or ANOVA was used, while the Wilcoxon rank-sum test and/or Mann–Whitney test for independent samples were used for non-normally distributed variables. For categorical variables, analysis was performed using the Pearson chi-square test or Fisher’s exact test, depending on the cell size. Univariate analysis results with at least one antigen of interest with *p* < 0.1 were included in the multivariate model.

A multivariate model for each vaccine-preventable disease was implemented to assess the risk factors associated with seroprotection, expressed as odds ratios (ORs) and corresponding 95% confidence intervals (CIs). Post hoc analysis on the subpopulation of subjects with undetectable viral loads (VL < 50 cp/mL) or with a CD4+ >200 cells/mm^3^ at the last dose of the primary series was implemented. The data were analyzed using R statistical software (Version 2025.05.1+513). Statistical significance was set at *p* < 0.05.

## 3. Results

### 3.1. Characteristics of the Study Population

A total of 86 PLHIV were followed in the Pediatric Infection Disease Unit for vertically acquired HIV infection. Of these, eighty-five were included in the analysis. One subject was excluded due to indeterminate vaccination status. The characteristics of the population are summarized in Table 1.

The median age was 24 years (IQR 16–31); all subjects were on ART at the time of the study, with a median duration of therapy of 21 years (IQR 14–24). Most of them had good immunovirological control at the time of analysis: the median CD4+ count was 793 cells/mL (IQR 614–1026), and viral load was suppressed in 92% of people.

The majority started ART at a median age of 53 months (IQR 12–118), with only 27% initiating treatment within the first year of life. A significant proportion (40%) had a stage 3 HIV infection according to the CDC classification in 2023.

### 3.2. Vaccination Coverage

To assess vaccination coverage, updated vaccination certificates were available for 80 of the 85 subjects. For the remaining five, vaccination status was determined based on anamnestic medical history. Additionally, six subjects in our cohort had HBV co-infection and were excluded from the HBV serological analysis only.

Regarding the primary immunization series, all subjects received at least one dose of tetanus during childhood. Only one subject did not receive any dose of diphtheria vaccine. A total of 73% of subjects in our cohort received a complete primary series for both diphtheria and tetanus. In total, 94% of people had complete vaccination coverage for HBV, 3.5% received fewer than three doses, and only 2.5% were not vaccinated.

Regarding live attenuated vaccines, 91% of subjects had received at least one dose of measles (with a complete cycle in 72%), 84% received mumps and rubella vaccines (with a full cycle in 64%), and only 24% received varicella vaccination (Table 2).

Among those with available vaccination certificates, only 22%, 37%, 40%, and 26% of subjects vaccinated for diphtheria/tetanus, measles, mumps/rubella, and hepatitis B, respectively, completed the entire primary vaccination series after initiating ART. In the remaining cases, immunization was performed before starting ART, or ART was initiated during the primary vaccination series. Regarding varicella, the complete vaccination series was entirely administered after the initiation of ART in 68% of cases (Table 3).

### 3.3. Seroprotection at Specific Time Points

**Figure 1:**
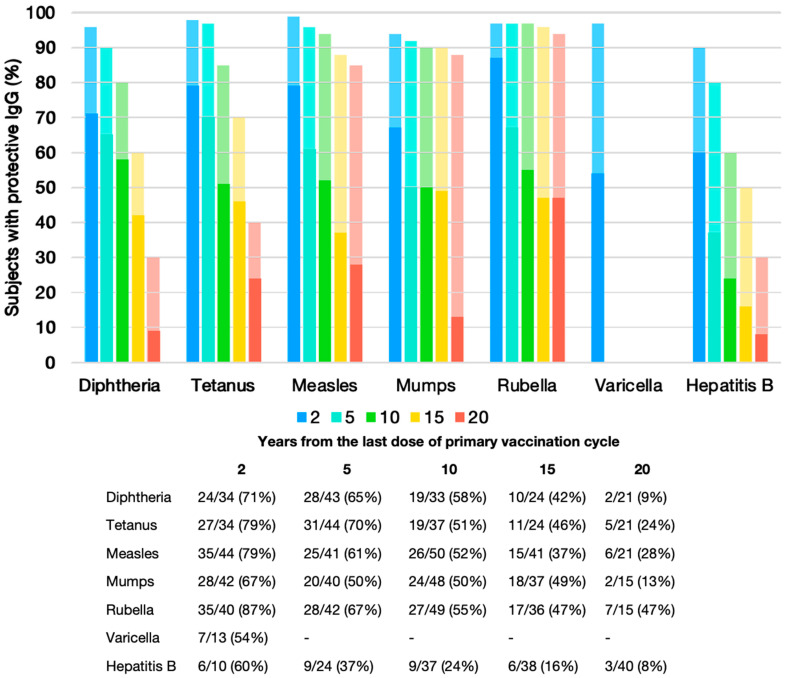
Proportion of PLHIV in our cohort (dark bars) and individuals from the general population (light bars) with protective IgG titers at different time intervals since completion of the primary vaccination series, by vaccine-preventable disease. Only time points with N > 10 are reported.

Two years after the last dose of the respective primary vaccination series, the proportion of subjects showing a protective level of antibodies was 71% for diphtheria, 79% for tetanus and for measles, 67% for mumps, 87% for rubella, 54% for varicella, and 60% for hepatitis B. Evaluation at subsequent time points revealed a gradual decline in protective antibody levels for all vaccine types as observation time increased, similar in what is reported for the general population in the literature (Figure 1 and Table S1 in Supplementary Material).

Overall, at the 5-year time point, approximately 50–70% of subjects had protective IgG values for diphtheria, tetanus, measles, mumps, rubella, and varicella. Excluding varicella due to limited data, protection declined to 50–58% at 10 years and 37–49% at 15 years. HBV antibodies decline more rapidly, reaching lower values, with only 37%, 24%, and 16% having protective antibodies at 5, 10, and 15 years, respectively. By the 20-year observation point, protective antibody levels were observed in only 24% of subjects vaccinated for tetanus, 9% for diphtheria, 28% for measles, 13% for mumps, 47% for rubella, and 8% for HBV. Prevalence of HIV-positive subjects with protective IgG titers in different years since the last dose of primary vaccination cycle by different vaccine-preventable diseases and by initiation of ART within the first 12 months of life is depicted in Figure S1 in the Supplementary Material. Higher median HIV viral load values at the time of administration of the last dose of the primary series of the different vaccine-preventable diseases were noted for non-seroprotected individuals (Figure S2 in the Supplementary Material).

### 3.4. Seroprotection Rates in 2023

Out of the 85 subjects, 67 had recent serological data (between 2022 and October 2023), 15 had partially recent data (with some serologies performed before 2022), and 3 subjects had serological data entirely performed before 2022. Serologies were considered only for subjects who had received at least one dose of the corresponding immunization, including booster doses.

**Figure 2:**
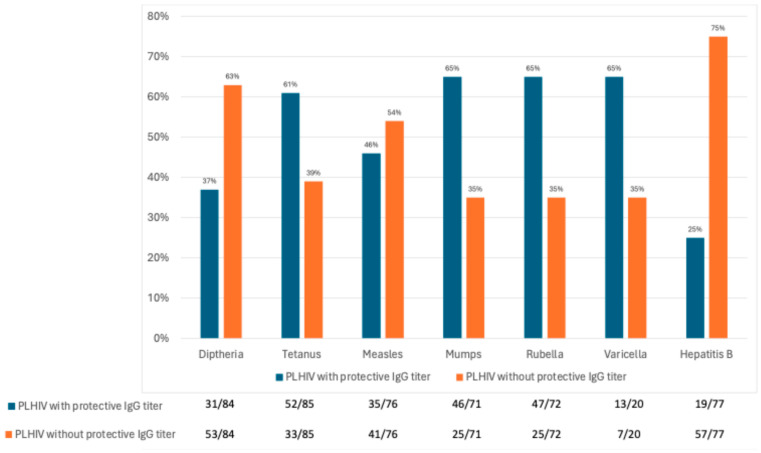
Seroprotection rates of different vaccine-preventable diseases in PLHIV in our cohort in 2023.

As shown in Figure 2, the proportion of individuals with protective antibody levels was 37% for diphtheria, 61% for tetanus, 46% for measles, 65% for mumps, 65% for rubella, 65% for varicella, and 25% for HBV.

### 3.5. Multivariate Analysis

Various factors were considered in the multivariate analysis, comparing seroprotected with non-seroprotected PLHIV. We found that for each additional year since the last dose of the primary series, the risk of not being seroprotected increased by 2% for diphtheria (OR: 0.98 (95%CI: 0.97–1.00); *p* value 0.028), tetanus (OR: 0.98 (95%CI: 0.96–0.99); *p* value = 0.003), and 4% for measles (OR: 0.96 (95%CI: 0.94–0.99); *p* value = 0.003) and rubella (OR: 0.96 (95%CI: 0.93–0.98); *p* value =0.001). No other factors showed a significant correlation with seroprotection, except for the CD4+ count and the viral load values at the time of the last dose of the primary vaccination series for mumps and the CDC3 stadium in 2023 for tetanus (Table 4). Restricting the analysis only to PLHIV with undetectable viral loads (VL < 50 cp/mL) or with a CD4+ > 200 cells/mm^3^ at the last dose of the primary series showed a similar effect trend regarding seroprotection (Table S2).

## 4. Discussion

Our findings demonstrate a progressive decline in protective IgG titers over time among PLHIV with vertically acquired infection, with a clear inverse relationship between antibody levels and the number of years since the last dose of the primary vaccination series. To assess the impact of time on antibody persistence while minimizing the confounding effect of age heterogeneity within the cohort, serological data were stratified by time intervals since the final dose of the primary immunization schedule. Two years post-vaccination, the proportion of seroprotected individuals was 71% for diphtheria, 79% for tetanus and measles, 67% for mumps, 87% for rubella, 54% for varicella, and 60% for hepatitis B. Compared with previously published data from the general population, these rates were consistently lower across all vaccinations considered [10,21,22,23,24,25,26,27,28,29].

Even when comparing data from our population at the 10-year time point with those reported in the literature for a similar population, we observed slightly lower values, particularly for MMR serologies. However, the values at the 5-year time point are more comparable. Indeed, a review conducted by Sutcliff et al. [11] highlighted considerable variability in seroprotection rates for vaccinations administered before ART initiation in HIV-positive children. Furthermore, Bekker et al. in their study [13] showed that, despite the initiation of effective therapy, 40% of children who were seroprotected at the start of ART lost protection for measles, 38% for mumps, and 11% for rubella after 2–3 years of ART.

In immunocompetent individuals, inactivated vaccines—such as those targeting diphtheria and tetanus—typically require periodic booster doses due to the physiological decline in antibody titers over time. Nevertheless, seroprotection generally remains above accepted thresholds for up to 10 years, with rates around 85% for tetanus and over 70% for diphtheria [21,22,23,24]. In contrast, our cohort consistently showed lower levels of seroprotection across all time points. At 5 years post-vaccination, one-third of participants lacked protective antibody titers, and approximately half were unprotected by 10 years, suggesting that booster doses may be warranted earlier than the standard decennial interval. These findings are consistent with previously published data in HIV-positive pediatric populations, where seroprotection at 5 years post-vaccination was observed in 70–80% for tetanus and 40–65% for diphtheria [4,12].

Unlike inactivated vaccines, live attenuated vaccines, such as MMR, are highly immunogenic in healthy individuals and generally elicit durable immunity, often persisting for decades or even a lifetime [30,31]. Although some decline in antibody titers has been documented over time, protective levels typically remain detectable in more than 85–90% of immunocompetent individuals 10–15 years after immunization [27,28,30]. In contrast, our cohort demonstrated markedly reduced seroprotection for MMR as early as two years after the final dose, with levels approximately 50% lower than those reported in healthy populations at 15 years. Between 2 and 5 years post-vaccination, seroprotection declined by roughly 20% across all three vaccine-preventable diseases. From 5 to 15 years, measles and rubella protection decreased by an additional 20–24%, while mumps seroprotection remained stable at approximately 50%. At 20 years, only 28% of measles-vaccinated individuals retained protective titers, while seroprotection dropped to 13% for mumps and stabilized at 47% for rubella. These observations are in line with findings from the meta-analysis by Kerneïs et al., which reported that 68% of primary responders retained protective antibody levels at 2 years, decreasing to 40% at 5 years [12].

In our cohort, only 54% at 2 years and 50% at 5 years showed protective antibodies for varicella, which is significantly lower than the values reported in the general population [30] but in line with another study conducted on HIV-infected children [12]. Indeed, in immunocompetent subjects, the vaccination induces a protective antibody titer in 97% of children vaccinated with one dose, which persists in more than 90% for at least 6 years after vaccination. However, the number of subjects in our cohort vaccinated for varicella was very low compared to the other vaccines. Indeed, it is important to consider that universal varicella vaccination was introduced in the Veneto region in 2006, as a non-mandatory stand-alone vaccine, with slow uptake. In 2017, it became part of the mandatory vaccination schedule for children attending schools, with a two-dose regimen at 12–15 months of age and 5 to 6 years of age, offered as a combination with the MMR vaccine [32,33]. This partially explains the low vaccine uptake in the population under examination.

Seroprotection against HBV was notably low in our cohort, particularly when compared with other vaccine-preventable diseases. At two years post-vaccination, only 60% of individuals had detectable protective antibody levels, a figure that declined sharply over time. By 10 years, just 25% retained seroprotection; at 15 and 20 years, this proportion further dropped to 16% and 7.5%, respectively.

While a physiological decline in antibody titers has also been described in healthy individuals, with reported seroprotection rates of approximately 82% at 5 years and 40–75% between 10 and 30 years post-vaccination [34], this does not typically translate into increased clinical susceptibility. Indeed, despite waning antibody levels, HBV vaccination is believed to confer long-term protection, largely due to its capacity to elicit a rapid anamnestic immune response upon viral exposure, facilitated by the long incubation period of the virus [31].

Nevertheless, the degree of seroprotection observed in our cohort appears significantly lower than expected. A meta-analysis by Kerneïs et al. reported that 61% of children maintained protective titers at 2 years post-vaccination, with levels decreasing to 30% at 5 years—values that remain higher than those observed in our study [12]. These findings suggest that vertically infected PLHIV may experience a more pronounced and rapid decline in HBV-specific immunity, potentially necessitating earlier or additional booster strategies.

Although all subjects in our cohort were on ART at the time of data collection and had achieved satisfactory CD4 count recovery at the last serological evaluation, the majority (65–80%) had been vaccinated before starting ART. The low levels of seroprotection observed in the short and, particularly, the long term, compared to the general population, suggest, as reported in the literature, that initiating ART does not halt the decline in previously acquired antibody responses. This indicates suboptimal recovery of the memory B and T-cell compartments [15,35].

Data on the long-term persistence of seroprotection (>5 years) in subjects with vertically transmitted HIV are scarce. An Italian study conducted on a cohort of 39 HIV-positive subjects with maternal–fetal transmission (Sticchi et al. [36]) including a wide age range (from 6 to 28 years), reported seroprevalence rates of 13% for diphtheria, 43% for tetanus, 31% for HBV, 20% for measles, 0% for mumps, and 27% for rubella measured at an average age of 18 years. When comparing these values to our data from the 10–15-year time points, we observed higher seroprotection in our cohort for all vaccinations analyzed, except for HBV, which aligns with other literature findings. One possible explanation for the higher seroprotection for MMR in our cohort could be the greater vaccination coverage for live attenuated vaccines (complete series in 74% of cases for measles and 66% for mumps and rubella), compared to 20% in the population described in the article by Sticchi et al. [36]. Furthermore, other immunological factors in this population, such as the timing of ART initiation and the percentage of subjects who completed the vaccination cycle during ART, might differ from ours and could have influenced the outcome; however, these factors were not reported.

Fewer than 30% of participants in our cohort completed the full primary vaccination series while receiving ART, with the notable exception of varicella, for which more than 60% of individuals were vaccinated during ART. This pattern likely reflects historical treatment timelines: combination ART became widely available only after 1996, meaning that individuals born before the mid-1990s typically did not initiate therapy until after the age of 10—by which time the primary vaccination schedule had already been completed. Conversely, varicella vaccination was introduced into the immunization program more recently, coinciding with earlier ART initiation in younger children and, consequently, better virological control during early childhood.

Completion of the vaccination series while on ART appears to enhance serological protection, particularly for diphtheria, measles, and rubella. Several studies have shown that ART initiation can restore responsiveness to novel antigens, even in individuals with advanced immunosuppression at diagnosis [11,12,15]. Nonetheless, although ART promotes antigen-specific immune recovery, vaccine-induced responses in PLHIV remain quantitatively and qualitatively inferior to those observed in the general population, suggesting persistent immune dysfunction despite treatment [10,12].

Multiple studies have examined predictors of vaccine responsiveness in ART-treated individuals, though results have been heterogeneous. In general, higher CD4+ T-cell counts and suppressed or low HIV viral load at the time of vaccination are associated with improved seroprotection, although this association is not universally observed. Better immunogenicity—particularly for diphtheria and tetanus—has also been linked to milder disease stage (CDC stage 1 or 2) and higher CD4+ levels at the time of the last vaccine dose [4,12]. In contrast, individuals with advanced disease may experience more profound and prolonged immune damage, which may impair the establishment of durable vaccine-induced immunity, even after immune reconstitution is achieved through ART [37].

In our multivariate analysis, we found that a higher number of CD4 cells at the time of vaccination correlated with a better vaccine outcome only for the mumps vaccine. However, due to the limited data in our study, this association cannot be considered strong.

In our analysis, a shorter time interval since the last vaccine dose was significantly associated with higher seroprotection rates, particularly for tetanus, diphtheria, and measles. Additionally, lower HIV viral load at the time of the final dose of the primary vaccination series appeared to be associated with improved antibody response to mumps. However, no clear association was observed between ART initiation before 12 months of age and vaccine-induced immunity.

These findings differ from previous reports suggesting that early ART initiation may enhance long-term vaccine response. Notably, studies have shown that initiating ART before the age of 12 months is associated with preserved antibody responses in up to 82% of measles-vaccinated and 92% of tetanus-vaccinated children at four-year follow-up. In contrast, those who initiated ART after 12 months demonstrated significantly lower protection rates (38–39%) despite comparable CD4+ T-cell counts and virological suppression at the time of booster administration [18].

HIV infection induces complex alterations in the immune system, leading to a progressive decline in CD4 T-cells and B lymphocytes. B-cell depletion, particularly of CD27+ memory B-cells, results in the progressive loss of the acquired memory repertoire [18]. HIV infection also causes continuous polyclonal B-cell hyperactivation, which exposes B-cells to early exhaustion, alters cytokine patterns, and induces functional changes in receptor expression. These changes shorten cell half-lives and reduce the ability to generate antigen-specific responses. In vitro studies have shown that, despite numerical increases in B and T lymphocytes during ART, there remains a degree of functional impairment, with shortened half-lives of memory B-cells and plasma cells [12,38]. These alterations are not restored by ART and may explain the persistent deficit in antibody and cellular responses to vaccinations, characterized by a primarily shorter duration of protection over time [11,18,38].

Immunological alterations induced by HIV appear early in infection [18,38]. Prolonged exposure to uncontrolled viral replication is strongly correlated with reduced reversibility of immune dysfunction and accelerated immunosenescence, even following ART initiation. This is underscored by evidence showing that initiation of ART during advanced stages of disease does not consistently lead to full restoration of CD4+ T-cell counts [39]. Children with vertically acquired HIV experience a more profound impact on immune function than those with horizontally acquired infection. This is largely attributable to the disruption of immune system development during critical periods of early life, resulting in altered maturation pathways and incomplete protection against vaccine-preventable diseases during the most vulnerable stages of infancy and early childhood [40].

Early initiation of ART, particularly within the first 12 months of life, appears to mitigate long-term HIV-induced immune damage, promoting both the functional development of the immune system and preservation of the memory B-cell compartment. This is exemplified by the findings of Pensieroso et al. [18], who demonstrated that HIV-infected children initiating ART before 12 months of age exhibited humoral responses indicative of intact memory B-cell function, with B-cell counts comparable to those observed in HIV-negative control groups.

This study has several limitations. First, the sample size was relatively small, in part due to stratification across multiple time points, which reduced the number of observations within each subgroup. Second, the retrospective design relied on pre-existing clinical and serological data extracted from both electronic and paper medical records, with data collected at heterogeneous and sometimes inconsistent time intervals. This led to occasional missing values and reduced standardization.

In addition, a subset of participants had initially been followed at other healthcare facilities or in their country of origin, which limited the availability of complete documentation and contributed to gaps in the dataset. Additionally, viral load assessments were systematically introduced at our center only from 1996 to 1997 onward. Consequently, for older participants—who received the primary vaccination series prior to HIV diagnosis and ART initiation—virological and immunological data such as CD4+ T-cell counts at the time of vaccination were not retrievable. Moreover, other critical variables that could have enhanced stratification—such as CD4+ nadir, adherence to ART, and longitudinal viral suppression—were inconsistently available across the cohort. The lack of these parameters limited our ability to fully characterize the relationship between immune status, treatment response, and vaccine-induced immunity.

Our cohort is heterogeneous in terms of origin and age, and vaccination schedules varied due to the differing immunogenicity of vaccine types and the use of different vaccination calendars, which could have influenced the vaccine outcomes. Lastly, our study uses antibody positivity as a surrogate marker of protection. However, the correlation with actual vaccine efficacy in this population is not well-studied [11,36], and we did not consider cellular response, as this was a retrospective study based on routine clinical practice data. Nevertheless, a strength of our study is the long-term follow-up, in some cases extending up to twenty years, in a specific cohort of subjects with vertically acquired infection.

## 5. Conclusions

Despite the initiation of ART, PLHIV who have vertically transmitted infections exhibit lower antibody levels against vaccine-preventable diseases compared to non-infected individuals. Therefore, strict adherence to the vaccination schedule and regular serological testing are crucial for PLHIV to ensure adequate protection against potentially harmful infections.

## Figures and Tables

**Table 1:** Demographic, immunovirological, and therapeutic characteristics of the PLHIV cohort. IQR: interquartile range; ART: antiretroviral therapy; VL: viral load; cART combination antiretroviral therapy; CDC Center for Disease Control and Prevention; *n*: number.

Demographic Characteristics	HIV-Positive Subjects (*n* = 85)
Sex	47 F (55%); 38 M (45%)
Age (years), median (IQR)	24 (16, 31)
Country of birth, *n* (%)	
Italy	41 (48%)
Africa	28 (33%)
Others	16 (19%)
HIV infection stage before starting ART, *n* (%)	
CDC 1	32 (38%)
CDC 2	21 (24%)
CDC 3	32 (38%)
HIV infection stage in 2023, *n* (%)	
CDC 1	30 (36%)
CDC 2	21 (24%)
CDC 3	34 (40%)
Undetectable VL, *n* (%)	78 (92%)
VL (cp/mL) among subjects with unsuppressed VL in 2023, median (IQR)	6410 (59, 133,000)
CD4+ cell/mm^3^ in 2023, median (IQR)	793 (614, 1026)
CD4+ percentage in 2023, median (IQR)	36.5 (30.5, 41.8)
Subjects in ART in 2023, *n* (%)	85 (100%)
ART duration in 2023 (years), median (IQR)	21 (14, 24)
Age at ART initiation (months), median (IQR)	53 (12, 118)
Initiation of ART within the first 12 months of life, *n* (%)	
Yes	23 (27%)
No	58 (68%)
Not available	4 (5%)

**Table 2:** Immunization coverage for the primary series, by vaccine and completion status.

	Subjects with a Completed Vaccine Schedule, *n* (%)	Subjects with an Incomplete Vaccine Schedule, *n* (%)	Subjects Without Any Dose of Vaccine, *n* (%)
Anti-Diphtheria *n* = 85	62 (73.0%)	22 (26.0%)	1 (1.0%)
Anti-Tetanus, *n* = 85	62 (73.0%)	23 (27.0%)	0 (0.0%)
Anti-Measles, *n* = 85	61 (72.0%)	16 (19.0%)	8 (9.0%)
Anti-Mumps, *n* = 85	54 (64.0%)	17 (20.0%)	14 (16.0%)
Anti-Rubella, *n* = 85	54 (64.0%)	18 (20.0%)	13 (16.0%)
Anti-Varicella, *n* = 85	16 (19.0%)	4 (5.0%)	65 (76.0%)
Anti-Hepatitis B, *n* = 79	74 (94.0%)	3 (3.5%)	2 (2.5%)

**Table 3:** Timing of diphtheria/tetanus, measles, mumps/rubella, varicella, and hepatitis B primary vaccination series in relation to ART initiation in PLHIV with available data on immunization and ART initiation.

	Anti- Diphtheria and Anti- Tetanus N = 76	Anti- Measles N = 67	Anti-Mumps and Rubella N = 62	Anti- Varicella N = 19	Anti- Hepatitis B N = 68
Subjects with primary vaccination series while on ART					
only the last dose, *n* (%)	30 (40%)	23 (34%)	23 (37%)	3 (16%)	14 (21%)
complete series, *n* (%)	17 (22%)	25 (37%)	25 (40%)	13 (68%)	18 (26%)
Subjects with no doses of primary vaccination series while on ART, *n* (%)	29 (38%)	19 (28%)	14 (23%)	3 (16%)	36 (53%)

**Table 4:** Odds ratios with 95% confidence intervals of seroprotection related to different vaccine-preventable diseases.

	Diphtheria		Tetanus		Measles	
	OR (95% CI)	*p* Value	OR (95% CI)	*p* Value	OR (95% CI)	*p* Value
ART start within 12 months of life	0.97 (0.72–1.30)	0.825	1.04 (0.79–1.37)	0.759	1.24 (0.89–1.73)	0.206
CDC3 stadium in 2023	0.85 (0.67–1.09)	0.213	0.78 (0.62–0.98)	0.039	1.12 (0.84–1.50)	0.444
CD4 for the last dose of the primary series	1.00 (1.00–1.00)	0.289	1.00 (1.00–1.00)	0.182	1.00 (1.00–1.00)	0.079
Viral load groups for the last dose of the primary series						
<50 copies/mL (ref)	ref		ref		ref	
50 < copies/mL < 200	0.76 (0.50–1.17)	0.223	0.73 (0.49–1.09)	0.128	1.18 (0.67–2.07)	0.574
>200 copies/mL	0.85 (0.62–1.16)	0.314	1.06 (0.79–1.43)	0.683	1.00 (0.72–1.40)	0.999
Years from the last dose of the primary series	0.98 (0.97–1.00)	0.028	0.98 (0.96–0.99)	0.003	0.96 (0.94–0.99)	0.003
Complete vaccination	0.92 (0.71–1.13)	0.547	1.10 (0.86–1.42)	0.454	0.76 (0.52–1.10)	0.153
Male sex	0.92 (0.71–1.13)	0.377	1.17 (0.94–1.45)	0.173	1.02 (0.77–1.34)	0.914
	Mumps		Rubella		HBV	
	OR (95% CI)	*p* value	OR (95% CI)	*p* value	OR (95% CI)	*p* value
ART start within 12 months of life	0.97 (0.72–1.31)	0.843	1.12 (0.81–1.55)	0.492	1.19 (0.83–1.71)	0.343
CDC3 stadium in 2023	1.00 (0.76–1.30)	0.971	0.85 (0.60–1.20)	0.358	1.07 (0.80–1.44)	0.635
CD4 for the last dose of the primary series	0.99 (0.99–1.00)	0.017	1.08 (0.81–1.46)	0.595	1.00 (1.00–1.00)	0.964
Viral load groups for the last dose of the primary series						
<50 copies/mL (ref)	ref		ref		ref	
50 < copies/mL < 200	0.53 (0.32–0.86)	0.016	1.00 (1.00–1.00)	0.97	0.97 (0.62–1.51)	0.878
>200 copies/mL	0.70 (0.52–0.95)	0.026	1.47 (0.85–2.55)	0.176	0.76 (0.52–1.11)	0.161
Years from the last dose of the primary series	0.98 (0.96–1.00)	0.086	0.96 (0.93–0.98)	0.001	0.99 (0.97–1.01)	0.271
Complete vaccination	0.81 (0.58–1.13)	0.221	1.09 (0.79–1.52)	0.605	0.60 (0.35–1.03)	0.075
Male sex	1.14 (0.88–1.48)	0.335	0.99 (0.75–1.31)	0.954	0.82 (0.62–1.08)	0.159

## Data Availability

The data used in this study cannot be made publicly available due to Italian data protection laws. The anonymized datasets generated during and/or analyzed during the current study can be provided on reasonable request from the corresponding author after written approval by the Ethic Committee.

## Supplementary Materials

The following supporting information can be downloaded at: https://www.mdpi.com/article/10.3390/vaccines13080871/s1, 1. Reference ranges for serologies. 2. Table S1. Proportion of PLHIV with protective, incomplete, or negative IgG levels, divided by vaccine-preventable disease and time elapsed since the last dose of the respective primary vaccination series. 3. Figure S1. Prevalence of HIV-positive subjects with protective IgG titers in different years since the last dose of primary vaccination series by vaccine-preventable disease and by initiation of ART within the first 12 months of life (plain box = ART initiated, dotted box = ART not initiated). Only seroprotection reported for N > 10 is shown. 4. Figure S2. Box and whiskers plots of HIV viral load values at the time of administration of the last dose of the primary series of the different vaccine-preventable diseases stratified by seroprotected (P) and non-seroprotected (N) PLHIV individuals. 5. Table S2. Odds ratio with 95% confidence intervals of seroprotection related to different vaccine-preventable diseases of the subgroup of PLHIV with undetectable viral loads (VL < 50cp/mL) or with a CD4+ >200 cells/mm^3^ at the last dose of the primary series.

## Abbreviations

The following abbreviations are used in this manuscript:

ART	Antiretroviral Therapy
cART	Combination Antiretroviral Therapy
VL	Viral Load
CI	Confidence Interval
CDC	Center for Disease Control and Prevention
ELISA	Enzyme Linked Immunosorbent Assay
IQR	Interquartile Range
MMRV	Measles, Mumps, Rubella, and Varicella
OR	Odds Ratio
PLHIV	People Living with HIV
RT-PCR	Real Time—Polymerase Chain Reaction
