# Supplementary material for: Immune Response to Childhood Vaccination in Vertically Infected People Living with HIV: A Long-Term Evaluation
Source: Vaccines (Basel). 2025 Aug 16;13(8):871. doi: 10.3390/vaccines13080871 (PMC12390248; doi:10.3390/vaccines13080871)
Supplement: Supplementary file 1 [file vaccines-13-00871-s001.zip › vaccines-3769573-supplementary.pdf]

Supplementary material

1. Reference ranges for serologies

2. Table S1. Proportion of PLHIV with protective, incomplete, or negative IgG levels, divided by vaccine-preventable disease and time elapsed since the last dose of the respective primary vaccination series.

3. Figure S1. Prevalence of HIV-positive subjects with protective IgG titres in different years since the last dose of primary vaccination series by vaccine-preventable disease and by initiation of ART within the first 12 months of life (plain box=ART initiated, dotted box=ART not initiated). Only seroprotection reported for N>10 is shown.

4. Figure S2. Box and whiskers plots of HIV viral load values at the time of administration of the last dose of the primary series of the different vaccine-preventable disease stratified by seroprotected (P) and non seroprotected (N) PLHIV individuals.

5. Table S2. Odds ratio with 95% confidence intervals of seroprotection related to different vaccine-preventable diseases of the subgroup of PLHIV with undetectable viral loads (VL<50cp/mL) or with a CD4+ >200 cells/mm^3 at the last dose of the primary series.

Reference ranges for serologies

Reference ranges for interpreting quantitative results, consistent with published studies[1]:

- IgG anti-diphtheria toxin (IU/mL): <0.01 negative; 0.01 – 0.09 incomplete response; ≥ 0.1 protective.
- IgG anti-tetanus toxin (IU/mL): <0.1 negative; 0.1 – 0.5 partial protection (considered as positive); >0.5 positive with good immune coverage
- IgG anti-measles (IU/mL): <200 negative; 200 – 274 doubtful; > 274 positive
- IgG anti-mumps (qualitative data): negative; positive
- IgG anti-rubella virus (IU/mL): <5 negative; 5 – 9.9 doubtful; ≥ 10 positive
- IgG anti-varicella zoster virus (IU/mL): <135 negative; 135-165 doubtful; > 165 positive
- Anti-HbsAG antibodies (IU/L): < 10 negative; ≥ 10 positive

Table S1. Proportion of PLHIV with protective, incomplete, or negative IgG levels, divided by vaccine preventable disease and time elapsed since the last dose of the respective primary vaccination series.

	Seroprevalence at time points				
	**T1**	**T2**	**T3**	**T4**	**T5**
Diphtheria, IgG	n= 34	n= 43	n = 33	n = 24	n = 21
Protective	24 (71%)	28 (65%)	19 (58%)	10 (42%)	2 (9%)
Incomplete	9 (26%)	11 (26%)	13 (39%)	12 (50%)	17 (81%)
Negative	1 (3%)	4 (9%)	1 (3%)	2 (8%)	2 (10%)
Tetanus, IgG	n = 34	n = 44	n = 37	n = 24	n = 21
Protective	27 (79%)	31 (70%)	19 (51%)	11 (46%)	5 (24%)
Incomplete	7 (21%)	1 (2%)	0 (0%)	0 (0%)	0 (0%)
Negative	0 (0%)	12 (27%)	18 (49%)	13 (54%)	16 (76%)
Measles, IgG	n = 44	n =41	n = 50	n = 41	n = 21
Protective	35 (79%)	25 (61%)	26 (52%)	15 (37%)	6 (28%)
Doubtful	4 (9%)	2 (5%)	2 (4%)	0 (0%)	1 (5%)
Negative	5 (11%)	14 (34%)	22 (44%)	26 (63%)	14 (67%)
Mumps, IgG	n = 42	n = 40	n = 48	n = 37	n = 15
Protective	28 (67%)	20 (50%)	24 (50%)	18 (49%)	2 (13%)
Doubtful	2 (5%)	4 (10%)	2 (4%)	1 (3%)	2 (13%)
Negative	12 (29%)	16 (40%)	22 (46%)	18 (49%)	11 (73%)
Rubella, IgG	n =40	n = 42	n = 49	n = 36	n = 15
Protective	35 (87%)	28 (67%)	27 (55%)	17 (47%)	7 (47%)
Doubtful	0 (0%)	1 (2%)	1 (2%)	1 (3%)	1 (7%)

Negative	5 (13%)	13 (31%)	21 (43%)	18 (50%)	7 (47%)
Varicella, IgG	n = 13	n =8	n=0	n = 0	n = 0
Protective	7 (54%)	4 (50%)			
Doubtful	1 (8%)	1 (13%)			
Negative	5 (38%)	3 (38%)			
HBV, IgG	n = 10	n = 24	n = 37	n = 38	n = 40
Protective	6 (60%)	9 (37%)	9 (24%)	6 (16%)	3 (8%)
Negative	4 (40%)	15 (63%)	28 (76%)	32 (84%)	37 (92%)

T1: 2 years; T2: 5 years +/- 1 year; T3: 10 years +/- 2 years; T4: 15 years +/- 2 years; T5: 20 years +/- 2 years; HBV: Hepatitis B virus

Figure S1. Prevalence of HIV-positive subjects with protective IgG titres in different years since the last dose of primary vaccination series by vaccine preventable disease and by initiation of ART within the first 12 months of life (plain box=ART initiated, dotted box=ART not initiated). Only seroprotection reported for N>10 is shown.

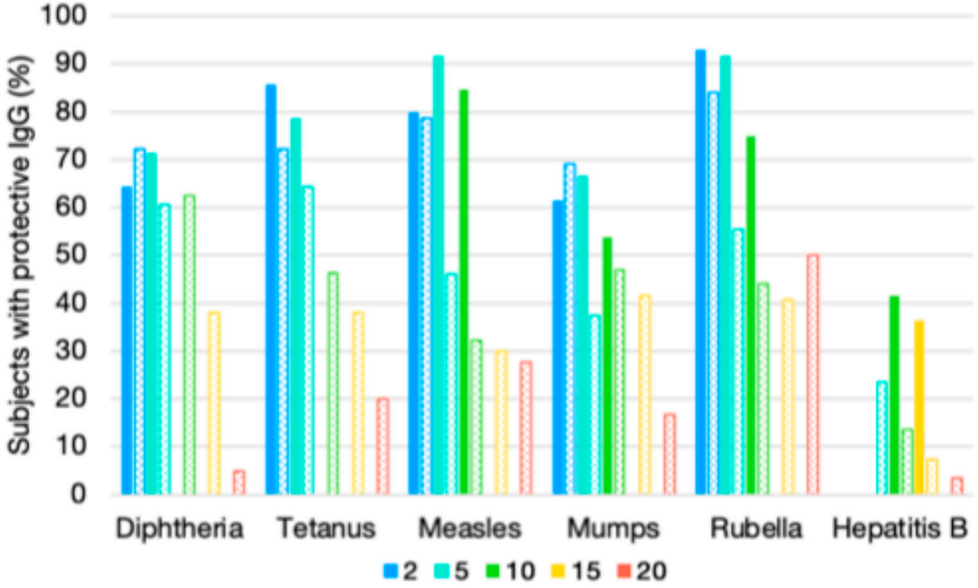

Years from the last dose of primary vaccination cycle

	ART	No ART	ART	No ART	ART	No ART	ART	No ART	ART	No ART
	2		5		10		15		20	
**Diphtheria**	9/14 (64%)	13/18 (72%)	10/14 (71%)	17/28 (61%)		15/24 (63%)		8/21 (38%)		1/20 (5%)
**Tetanus**	12/14 (86%)	13/18 (72%)	11/14 (79%)	18/28 (64%)		13/28 (46%)		8/21 (38%)		4/20 (20%)
**Measles**	12/15 (80%)	22/28 (79%)	11/12 (92%)	12/26 (46%)	11/13 (85%)	10/31 (32%)		9/30 (30%)		5/18 (28%)
**Mumps**	8/13 (62%)	18/26 (69%)	8/12 (67%)	9/24 (38%)	7/13 (54%)	15/32 (47%)		10/24 (42%)		2/12 (17%)
**Rubella**	13/14 (93%)	21/25 (84%)	11/12 (92%)	15/27 (56%)	9/12 (75%)	15/34 (44%)		11/27 (41%)		6/12 (50%)
**Hepatitis B**				4/17 (24%)	5/12 (42%)	3/22 (14%)	4/11 (36%)	2/27 (7%)		1/29 (3%)

Figure S2. Box and whiskers plots of HIV viral load values at the time of administration of the last dose of the primary series of the different vaccine preventable disease stratified by seroprotected (P) and non-seroprotected (N) PLHIV individuals.

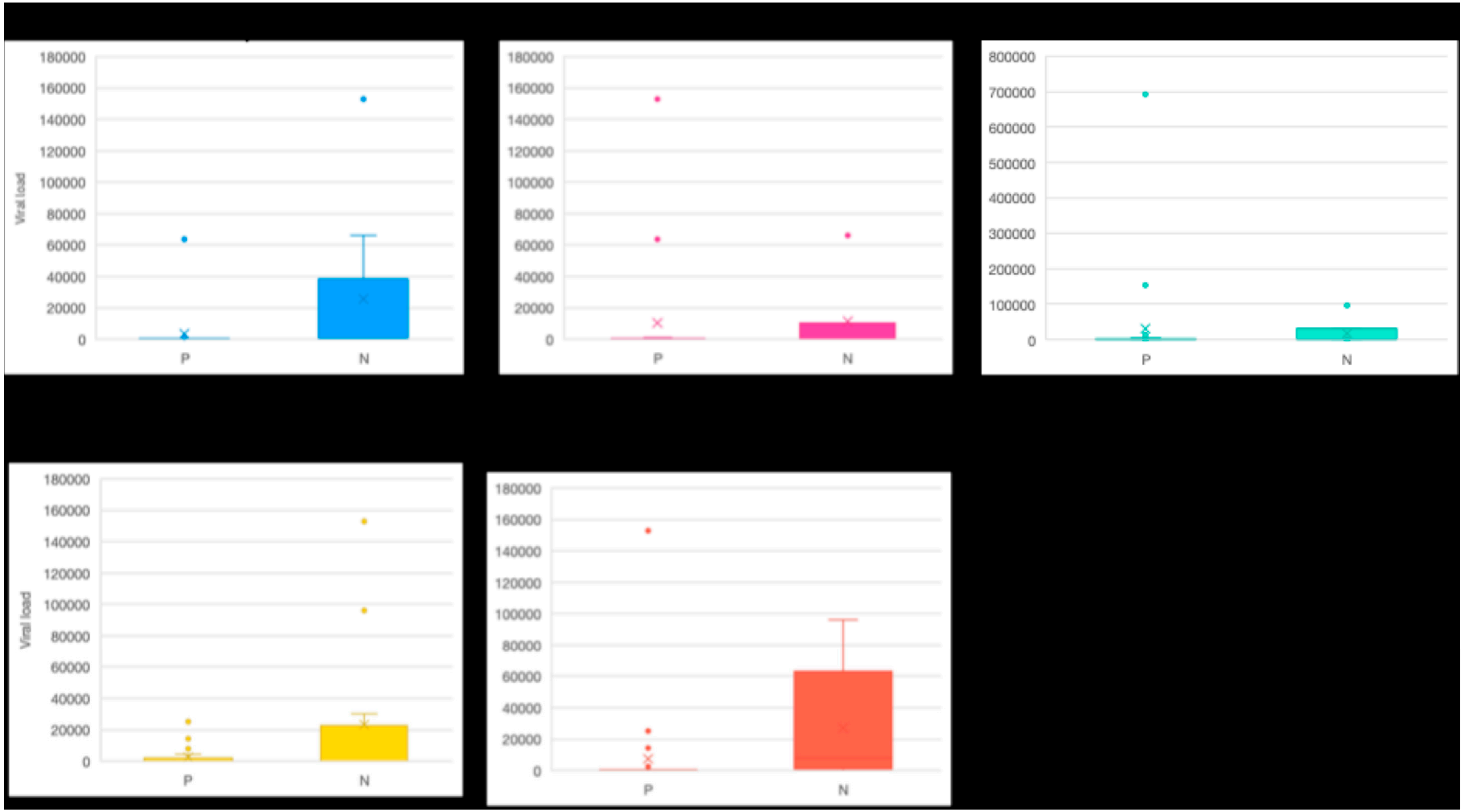

Table S2. Odds ratio with 95% confidence intervals of seroprotection related to different vaccine-preventable diseases of the subgroup of PLHIV with undetectable viral loads (VL<50cp/mL) or with a CD4+ >200 cells/mm³ at the last dose of the primary series.

	Diphteria		Tetanus		Measles	
	OR (95% CI)	p value	OR (95% CI)	p value	OR (95% CI)	p value
ART start within 12 months of life	0.99 (0.72 -1.36)	0.955	1.13 (0.85 - 1.49)	0.415	1.53 (0.89 -2.63)	0.151
CDC3 stadium in 2023	0.85 (0.59 -1.23)	0.401	0.83 (0.60 - 1.14)	0.256	0.90 (0.51 -1.60)	0.723
CD4 for the last dose of the primary series	1.00 (1.00 -1.00)	0.087	1.00 (1.00 - 1.00)	0.363	1.00 (1.00 -1.00)	0.481
Years from the last dose of the primary series	0.97 (0.95 -1.00)	0.066	0.98 (0.96 - 1.00)	0.107	0.97 (0.93 -1.02)	0.321
Complete vaccination	0.93 (0.65 -1.33)	0.697	1.12 (0.82 - 1.55)	0.476	1.12 (0.57 -2.19)	0.742
Male sex	0.92 (0.66 -1.28)	0.614	1.00 (0.75 - 1.35)	0.982	0.77 (0.46 -1.28)	0.332
	Mumps		Rubella		HBV	
	OR (95% CI)	p value	OR (95% CI)	p value	OR (95% CI)	p value
ART start within 12 months of life	1.21 (0.92 -1.60)	0.198	1.31 (0.90 - 1.90)	0.183	1.79 (0.71 -4.56)	0.307
CDC3 stadium in 2023	1.02 (0.77 -1.37)	0.879	1.00 (1.00 - 1.00)	0.917	0.77 (0.43 -1.36)	0.428
CD4 for the last dose of the primary series	1.00 (1.00 -1.00)	0.230	1.18 (0.79 - 1.76)	0.436	1.00 (1.00 -1.00)	0.671
Years from the last dose of the primary series	1.00 (0.98 -1.02)	0.985	0.96 (0.93 - 0.99)	0.030	0.97 (0.92 -1.02)	0.348
Complete vaccination	0.88 (0.63 -1.24)	0.482	0.88 (0.55 - 1.40)	0.592	1.51 (0.46 -4.94)	0.542

Male sex	0.88 (0.68 -1.13)	0.323	0.84 (0.60 - 1.17)	0.317	0.92 (0.4 -2.12)	0.86
